# Changes in dive behavior during naval sonar exposure in killer whales, long-finned pilot whales, and sperm whales

**DOI:** 10.3389/fphys.2012.00400

**Published:** 2012-10-11

**Authors:** L. D. Sivle, P. H. Kvadsheim, A. Fahlman, F. P. A. Lam, P. L. Tyack, P. J. O. Miller

**Affiliations:** ^1^Institute of Marine ResearchBergen, Norway; ^2^Norwegian Defence Research Establishment (FFI), Maritime Systems DivisionHorten, Norway; ^3^Woods Hole Oceanographic InstitutionWoods Hole, MA, USA; ^4^Department of Life Sciences, Texas A&M Corpus ChristiTexas, TX, USA; ^5^Netherlands Organisation for Applied Scientific Research (TNO), Acoustics and Sonar Research GroupThe Hague, Netherlands; ^6^Sea Mammal Research Unit, University of St. AndrewsSt. Andrews, UK

**Keywords:** marine mammal, dive, sonar

## Abstract

Anthropogenic underwater sound in the environment might potentially affect the behavior of marine mammals enough to have an impact on their reproduction and survival. Diving behavior of four killer whales (*Orcinus orca*), seven long-finned pilot whales (*Globicephala melas*), and four sperm whales (*Physeter macrocephalus*) was studied during controlled exposures to naval sonar [low frequency active sonar (LFAS): 1–2 kHz and mid frequency active sonar (MFAS): 6–7 kHz] during three field seasons (2006–2009). Diving behavior was monitored before, during and after sonar exposure using an archival tag placed on the animal with suction cups. The tag recorded the animal's vertical movement, and additional data on horizontal movement and vocalizations were used to determine behavioral modes. Killer whales that were conducting deep dives at sonar onset changed abruptly to shallow diving (ShD) during LFAS, while killer whales conducting deep dives at the onset of MFAS did not alter dive mode. When in ShD mode at sonar onset, killer whales did not change their diving behavior. Pilot and sperm whales performed normal deep dives (NDD) during MFAS exposure. During LFAS exposures, long-finned pilot whales mostly performed fewer deep dives and some sperm whales performed shallower and shorter dives. Acoustic recording data presented previously indicates that deep diving (DD) is associated with feeding. Therefore, the observed changes in dive behavior of the three species could potentially reduce the foraging efficiency of the affected animals.

## Introduction

Our understanding of the effects of military sonars on marine mammals has increased in the past decades (Richardson et al., 1995; Nowacek et al., 2007), but large gaps of knowledge still exist (Nowacek et al., 2007). Attention has focused on beaked whales due to several stranding events coinciding in time and space with military sonar operations (e.g., Simmonds and Lopez-jurado, 1991; Frantzis, 1998; Jepson et al., 2003; Cox et al., 2006; D'Amico et al., 2009). It has been suggested that these strandings are associated with a change in dive behavior which leads to development of tissue nitrogen gas bubbles and symptoms related to decompression sickness (DCS) (Jepson et al., 2003). Cetaceans often are reported to respond to anthropogenic noise with avoidance (e.g., Morton and Symonds, 2002; Olesiuk et al., 2002; Kastelein et al., 2008a,b; Tyack, 2008), either by horizontally swimming away from the sound source (Nowacek et al., 2004; Lusseau, 2009; Miller et al., 2011) or vertically, by a change in diving behavior (Miller et al., 2009). Such behavioral responses may protect the animals from direct physical injuries such as hearing impairment, but are likely to involve costs of leaving preferred habitat, costs of increased energy of locomotion as well as reduced feeding or higher risks of predation, etc. (Lusseau, 2009). Since cetaceans spend a significant amount of their time submerged, almost all behavioral responses are expected to result in a change in dive pattern. Cetaceans find their prey at depth, but need to return to the surface to breathe. An optimal foraging dive should minimize the energetic cost of traveling to the depth of the prey and maximize the energetic intake (Kramer, 1988). A change in diving behavior may hence have potential consequences involving ecological effects such as reduced foraging efficiency as well as potential physiological consequences such as DCS (Kvadsheim et al., 2012).

In this paper, we investigate whether and how whales changed their diving behavior during exposures to naval sonar sounds. The diving behavior before and during sonar exposure was studied here for three odontocete species with very different natural diving behavior; the deep diving (DD) sperm whale (*Physeter macrocephalus*), the intermediate diving pilot whale (*Globicephala melas*) and the shallow diving (ShD) killer whale (*Orcinus orca*).

## Materials and methods

Data were collected during three field periods; November 2006, May/June 2008 and May/June 2009, in the Northern Norwegian Sea. The November season comprised two experiments with killer whales, the rest of the experiments were all conducted in May/June. All field experiments were conducted with the FFI research vessel “R/V H.U. Sverdrup II,” and with a smaller vessel, an outboard workboat (2006) or “M/S Strønstad” (2008, 2009) as an observation vessel. Whales were tagged with archival sensor packages recording their diving behavior (time versus depth) as well as received sound before, during and after sonar transmissions. Animal experiments were conducted under permits issued by the Norwegian Animal Research Authority (permits no 2004/20607 and S-2007/61201), and in compliance with ethical use of animals in experimentation. The research protocols were also approved by the University of St. Andrews Animal Welfare and Ethics Committee as well as the Woods Hole Oceanographic Institutional Animal Care and Use Committee.

### Tag and sonar equipment

Data on dive behavior were collected by dtags (digital tags, Johnson and Tyack, 2003) attached to the back of the whales by suction cups. These multi sensor tags include stereo hydrophones, a pressure sensor, 3-axis accelerometers, 3-axis magnetometer and a VHF radio transmitter. In this study we have focused on dive behavior. Data on vocal behavior and horizontal movements are also collected by the tag, and such data are reported in Miller et al. (2011).

After a period of 1–7 h collecting baseline behavioral data, sonar signals were transmitted to the whales using the Royal Netherlands Navy's multipurpose acoustic source SOCRATES (Sonar CalibRAtion and TESting, Netherlands Organization for Applied Scientific Research (TNO), The Hague, The Netherlands). Two different signal frequency bands were used; low frequency active sonar (LFAS, 1–2 kHz) and mid frequency active sonar (MFAS, 6–7 kHz) either as frequency modulated hyperbolic upsweep or downsweep signals. Maximum source levels varied from 197–214 dB re 1 μPa at 1 m with 1 s pulse duration and 20 s inter pulse intervals (duty cycle of 5%). A 10 min ramp up was conducted before full power transmission. Further details of the experimental procedures can be found in Miller et al. (2011) and Kvadsheim et al. (2009).

### Experimental procedure

Whales were located by visual observers or towed hydrophone arrays, and tagged from a small boat. The experimental procedure was as follows: (1) tag 1 or 2 whales in a group, (2) post-tagging observation period to collect data on baseline behavior after recovery from tagging, (3) conduct a Controlled Exposure Experiment (CEE) which consisted of 1–4 source vessel approach exposure sessions, (4) collect post-exposure data, and (5) recover tags upon release for analysis. The tag was attached to the whale using a long carbon fiber pole or a pneumatic tag launching system (Aerial Rocket Tagging System, ARTS) (Kvadsheim et al., 2009). The whales were tracked by VHF and visually during the entire period. After a baseline period of 1–7 h, the source vessel “Sverdrup,” towing the sonar source, started moving towards the whales from a distance of 5–8 km and at a speed of ~4 m/s while sequentially transmitting a series of LFAS, MFAS, or no sonar signals (Silent control). Only one single sonar sound type was presented during each vessel approach session, and the order of the sound types presented was changed between each whale subjects. “Sverdrup” adjusted its course to approach the whale until 1 km distant, at which point its course was fixed. The distant ramp-up and the approaching source ship resulted in an escalation of the received levels of sonar sound.

During each exposure session, visual observers ensured that a safety limit of 100 m was kept between any animal and the source, and a shut-down of the system would occur if any animal moved closer than this.

### Data analysis

Pressure recordings were converted to depth using calibration values for each tag device. A dive was defined as any submergence for longer than 10 seconds to a depth >1 m, and dive duration as the time period between two surfacings. Duration and maximum depth were identified for all dives.

#### Analysis of changes in dive behavior

Cetaceans conduct different types of dives during different activities such as feeding, socializing, and traveling. Deep dives likely represent foraging periods, while shallow dives may be either resting dives between deep dives, or relate to traveling or socializing. To separate deep and shallow dives, a log-frequency analysis was used (Sibley et al., 1990) for all three species. The log-frequency analysis followed the two-process model of Sibley et al. (1990), and the dive mode criteria were calculated by the formula given in Slater and Lester (1982) to minimize the number of misclassified dives (Miller et al., 2010).

To examine whether diving behavior changed during sonar exposure sessions, we compared diving behavior before and during sonar exposure sessions. Exposure sessions commenced at random with respect to the animals' behavioral mode and therefore dive behavior of each individual whale was compared with its own pre exposure (PRE) behavior (baseline) in the time period just before sonar onset. For each exposure (LFAS/MFAS/Silent) a period of equal duration as the exposure period was defined as PRE.

Changes in diving behavior were examined in three steps. The first step was to determine the dive mode of the animal in the PRE and Exposure periods using pre-defined criteria: (1) ShD, if the period contains only dives shallower than the log frequency criterion for that species. This ShD mode was usually associated with traveling. (2) DD, when the animal conducted a series of dives deeper than the log frequency criterion with only a few shallow dives between each deep dive. This DD mode was usually associated with foraging. (3) ShD with occasional deep dives (SoD), when the animal was diving shallow as for mode ShD, but then conducted one deep dive and returned to ShD. Such dives may be exploratory dives, e.g., to search for prey. Sperm whales are known to continuously conduct deep foraging dives with a few shallow dives between. However, during several of the exposures, deep dives (according to the log frequency criteria) appeared to be shallower than the regular foraging dives. DD for sperm whales were hence divided into normal deep dives (NDD) if they were within the average deep dive depth during baseline ±s.d, and unusually shallow deep dives (UsDD) if they were shallower than this range. Dives modes were categorized for the two periods (PRE and Exposure) to examine whether the animal changed its overall dive behavior during sonar exposure.

The second step was to measure three dive variables; dive duration, dive depth, and dive rate (number of dives/duration of period), for deep and shallow dives separately, and compare those between the PRE and Exposure period for each species and exposure type. The average and 95% confidence interval (1.96 standard error) were calculated and plotted for all these parameters to enable a comparison of the range of each of these parameters between the PRE and Exposure for each individual and exposure session.

Changes in depth and duration may reveal whether the nature of the dives changed during sonar exposure, while dive rate indicates whether the animal spent more or less time diving and is therefore an indication of changes in dive mode.

The third step involved an overall evaluation of each individual exposure, considering the dive mode as well as the range of depth, duration, and rate of the dives in the PRE and Exposure period, to define whether a change in dive behavior had occurred or not. Additionally, vocal records of echolocation and tailslaps during a dive (reported in Miller et al., 2011) were used to identify foraging dives. Each individual exposure was thus categorized as either a change in dive behavior in response to sonar or not.

## Results

### Normal diving behavior

**Figure 1:**
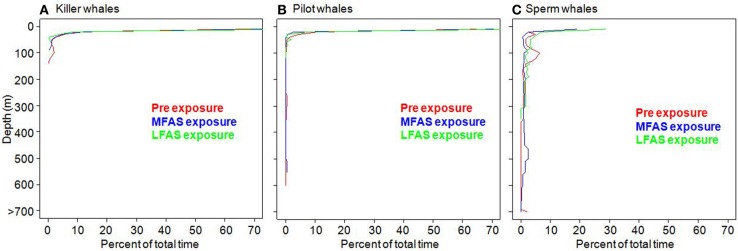
**Percent of total time spent at different depths in the water column during the different experimental conditions (LFAS, MFAS, PRE) for (A) killer whales, (B) long-finned pilot whales and (C) sperm whales**.

**Figure 2:**
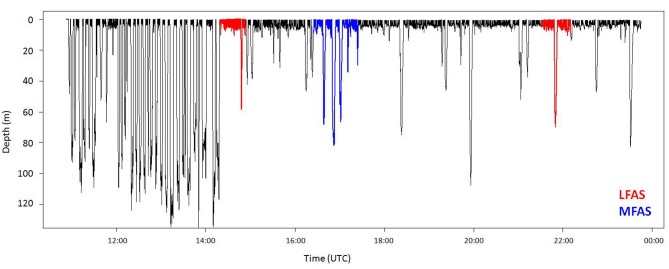
**Full dive record of killer whale oo09_144b**. The whale made deep dives prior to the first LFAS exposure, and stopped deep diving at onset (red). Both prior to and during MFAS exposure (blue), the killer whale conducted both shallow and deep dives. During the second LFAS exposure, the whale was shallow diving before sonar onset, and continued shallow diving with one occasional deep dive.

**Figure 3:**
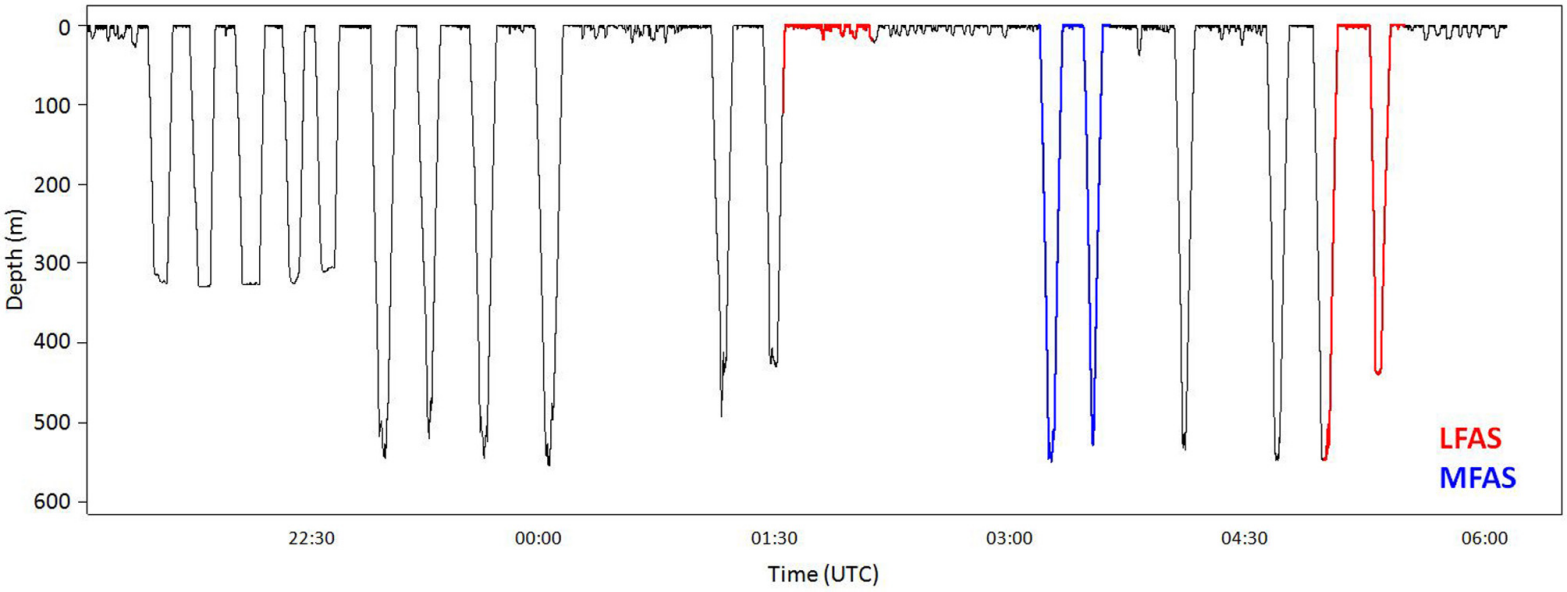
**Full dive record of pilot whale gm09_156b**. This record show typical pilot whale diving behavior, with periods of shallow diving, followed by bouts of deep dives. Before onset of the first LFAS exposure, the whale conducted two deep dives, but turned to shallow diving at LFAS exposure onset (red). The unusually low number of dives in this deep diving bout indicates that this may be an effect of the exposure. During MFAS exposure (blue), the whale conducted regular deep dives. During the second LFAS exposure, the whale continued deep diving.

**Figure 4:**
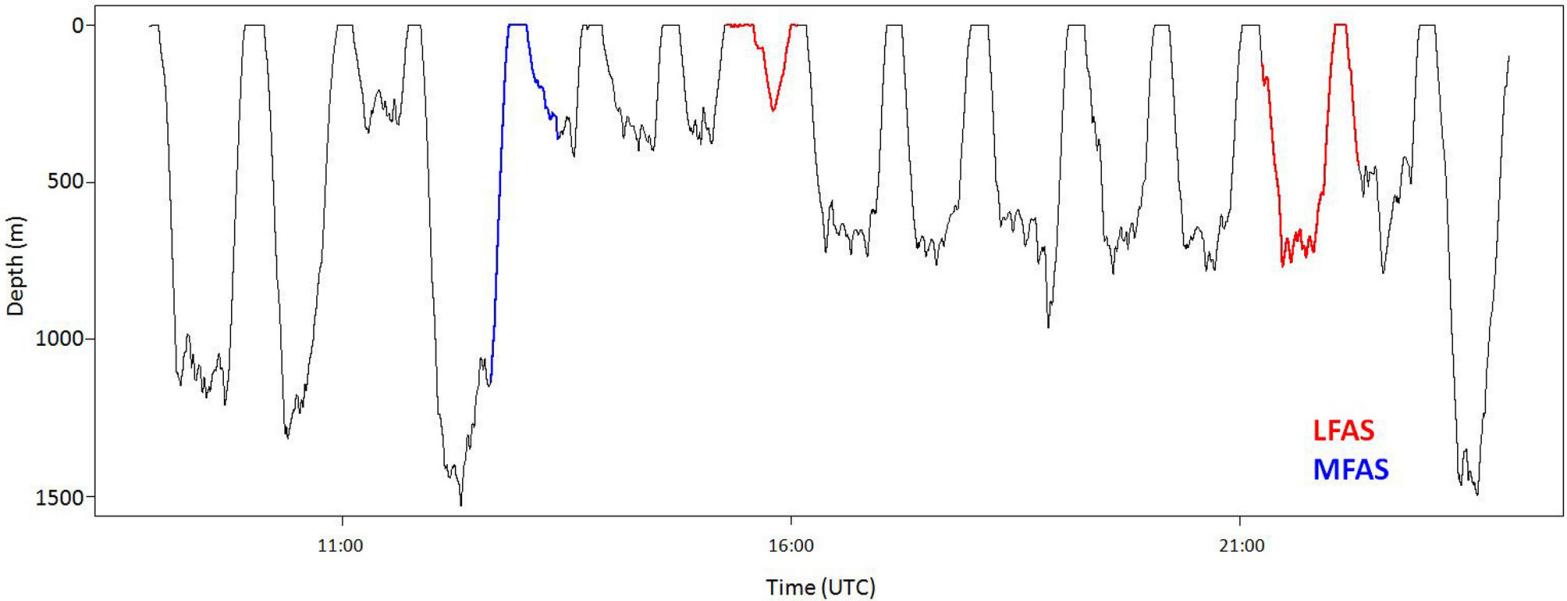
**Full dive record of sperm whale sw09_160a**. Record show typical sperm whale dive behavior (Teloni et al., 2008) with deep dives followed by a surface period. During MFAS exposure (blue), the animal switched from normal deep dives to shallower but still deep diving. During the first LFAS exposure (red), the animal conducted an unusually short and shallow dive after a long surface period. During the second LFAS exposure the whale conducted what appears as a normal deep dive.

Killer whales conducted most dives in the upper 50 m of the water column (Figure 1A), with periods of DD to a maximum depth of 140 m, alternating with periods of diving close to the surface (Figure 2). Long-finned pilot whales also spent the majority of their time in the upper 50 m (Figure 1B), separated by bouts of multiple deep foraging dives to 300–600 m (Figure 3). Sperm whales on the other hand spend more than 80% of their time deeper than 10 m (Figure 1), conducting long, deep foraging dives to 150–1500 m, each followed by a short surface period with a few shallow dives (Figure 4).

### Killer whales

The log frequency analysis estimated 21 m as the depth separating deep and shallow dives for killer whales. Deep dives tended to occur in bouts, with periods of ShD with only occasional single deep exploratory dives in between. An example of a killer whale dive record is shown in Figure 2.

#### LFAS

**Figure 5:**
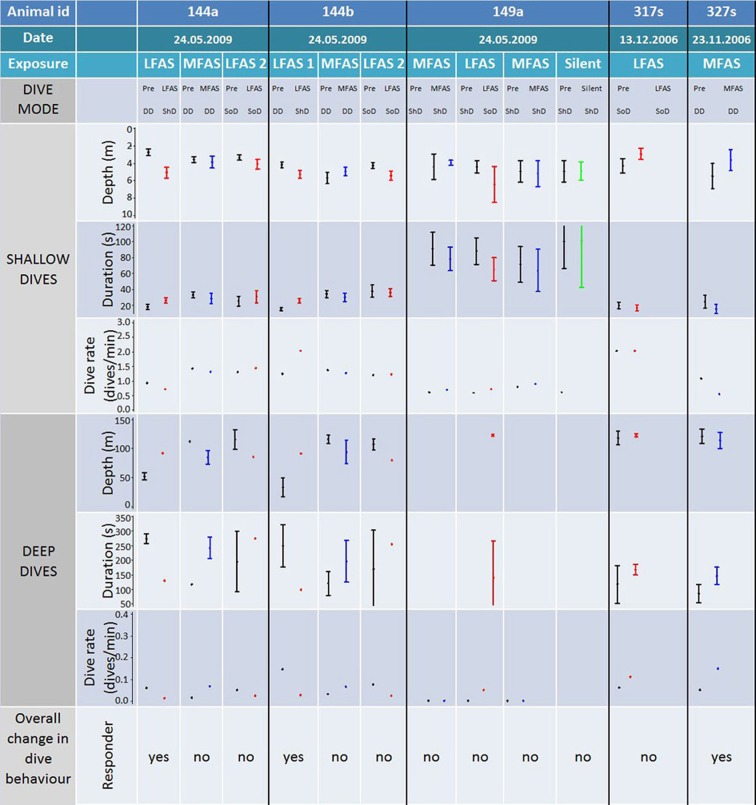
**Comparison of individual exposures to killer whales**. Figure shows animal id, exposure type, dive mode (during PRE and Exposure), as well as dive depth, duration, and rate for both shallow and deep dives. Log frequency analysis defined deep and shallow dives to be deeper and shallower than 21 m respectively for killer whales. Codes for dive modes are: deep diving (DD), shallow diving (ShD) and shallow diving with occasional deep dive (SoD). Bars show the 95% confidence interval. Dive rate for deep and shallow dives are number of dives in the PRE/Exposure period divided on the duration of the period, given as dives/min. All individual exposure sessions are classified either as a “responder” or not, based on the combined comparison of the depth, duration rate, and mode of the PRE and Exposure period.

A total of six LFAS exposures were conducted, with four different killer whales (Figure 5). Animals 144a and 144b were in DD mode at the onset of the first LFAS session. Visual inspection of the dive record indicates that they responded to the sonar by a clear change in behavior involving a switch to ShD (Figures 2, 5). For both animals in this exposure, depth, and duration of shallow dives increased compared to the PRE period (Figure 5). For the remaining four exposures, the animals (including 144a and 144b) were in ShD mode or SoD at the start of the exposure, and did not change their diving mode in response to the sonar (Figure 5). During these exposures there were no apparent differences in depth and duration of dives in the PRE and Exposure period (Figure 5).

#### MFAS

A total of 5 MFAS exposures were conducted with four different killer whales. None of these whales shifted dive mode during MFAS exposure (Figure 5). One whale (327 s) were conducting deep dives at sonar onset. This whale continued DD, but with a reduced rate for deep dives (Figure 5).

#### Silent control

Only one silent exposure was conducted. During this period there was no change in dive mode or any of the dive variables (Figure 5).

### Long-finned pilot whales

The log frequency analysis indicated 34 m as the separation depth for shallow vs. deep dives of long-finned pilot whales. Long-finned pilot whales had a distinct separation of periods of deep dives and periods of shallow dives. During DD periods, lasting typically 2–3 h, long-finned pilot whales conducted 5–10 dives to 300–600 m, each dive lasting 7–9 min. An example of a pilot whale dive record is shown in Figure 3.

#### LFAS

**Figure 6:**
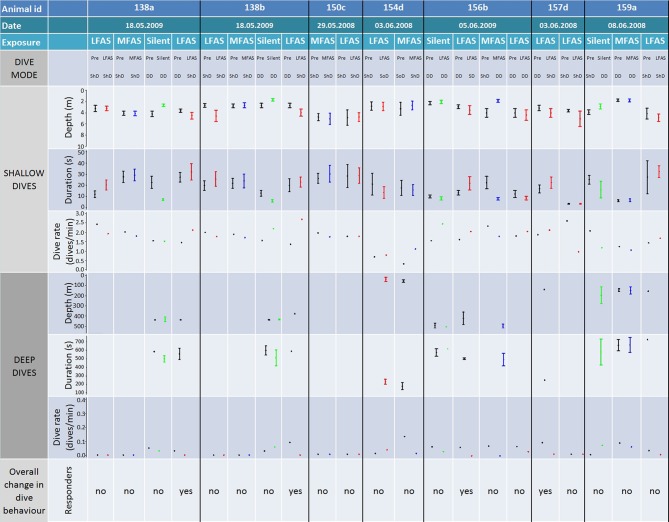
**Comparison of individual exposures to long-finned pilot whales**. Figure shows animal id, exposure type, dive mode (during PRE and Exposure), as well as dive depth, duration, and rate for both shallow and deep dives. Log frequency analysis defined deep and shallow dives to be deeper and shallower than 34 m respectively for killer whales. Codes for dive modes are: deep diving (DD), shallow diving (ShD) and shallow diving with occasional deep dive (SoD). Bars show the 95% confidence interval. Dive rate for deep and shallow dives are number of dives in the PRE/Exposure period divided on the duration of the period, given as dives pr min. All individual exposure sessions are classified either as a “responder” or not, based on the combined comparison of the depth, duration rate, and mode of the PRE and Exposure period.

A total of 11 LFAS exposures were conducted with seven different long-finned pilot whales. During 6 of the 11 exposures, the whales were in shallow dive mode at sonar onset, with all but one remaining in shallow mode throughout the exposure period. One whale (154d) switched to ShD with occasional dives greater than the 34 m criterion, but still shallower than 100 m depth. In the remaining five LFAS-exposures, the animals were in DD mode at sonar onset, with all but one (156b) shifting to shallow mode during LFAS exposure (Figure 6). These shifts were accompanied by increased dive rate for shallow dives and decreased rate for deep dives, with the shallow dives generally being deeper and of longer duration (Figure 6).

#### MFAS

A total of six MFAS exposures were conducted with six different long-finned pilot whales. Four of the six whales were in shallow dive mode at sonar onset, of which three stayed in shallow dive mode, and one initiated deep dive mode during MFAS exposure (156b). The remaining two whales were in deep dive mode at sonar onset, of which one continued DD (159a). The other (154d) had been conducting dives greater than 34 m though shallower than 100 m, and shifted to ShD during MFAS exposure (Figure 6).

#### Silent control

Four silent exposures were conducted with four different long-finned pilot whales. In three of four exposures, whales were in DD mode at onset and continued DD throughout the exposure period. One whale (159a) was in ShD mode at the onset, and initiated DD during exposure (Figure 6).

### Sperm whales

Sperm whales showed stereotyped dive behavior with regular DD to depths of 200–1500 m, with an average dive duration of 25 min followed by a period of 5–15 min of ShD close to the surface. The log-frequency analysis demonstrated 13 m as the separation depth for shallow vs. deep dives of sperm whales. The only dive mode observed for sperm whales was continuous deep dives with short inter-deep-dive intervals. An example of a sperm whale dive record is shown in Figure 4.

#### LFAS

**Figure 7:**
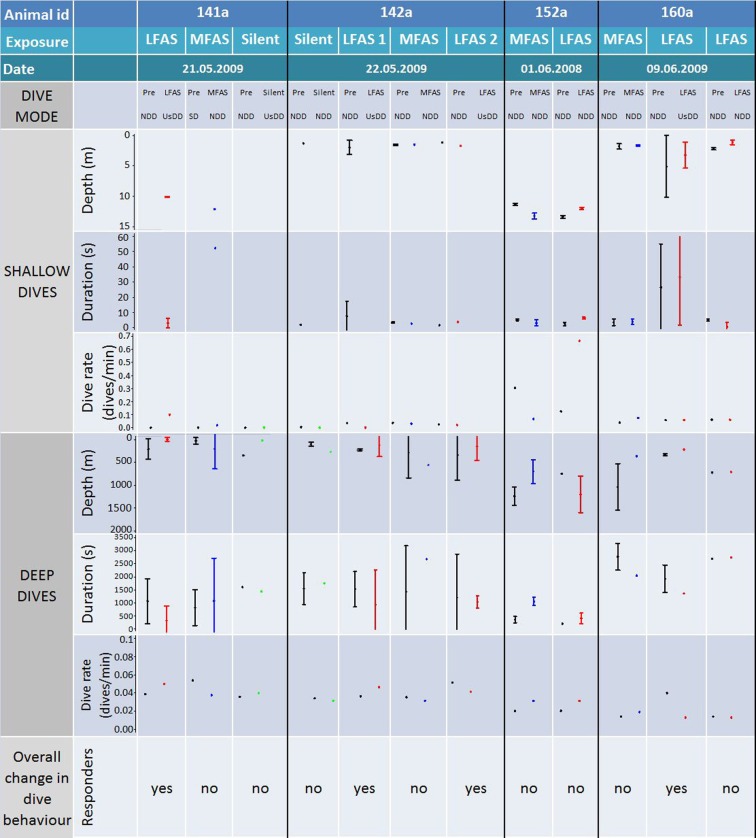
**Comparison of individual exposures to sperm whales**. Figure shows animal id, exposure type, dive mode (during PRE and Exposure), as well as dive depth, duration, and rate for both shallow and deep dives. Log frequency analysis defined deep and shallow dives to be deeper and shallower than 13 m respectively for killer whales. Codes for dive modes are: shallow diving (ShD), normal deep diving (NDD), and unusual shallow deep diving (UsDD). Bars show the 95% confidence interval. Dive rate for deep and shallow dives are number of dives in the PRE/Exposure period divided on the duration of the period, given as dives pr min. All individual exposure sessions are classified either as a “responder” or not, based on the combined comparison of the depth, duration rate, and mode of the PRE and Exposure period.

A total of six LFAS exposures were conducted with four different whales. Prior to all six exposures whales did normal deep diving (NDD). During four of the six LFAS exposures, whales shifted to UsDD, with all of these exposure deep dives being on average shallower and of shorter duration then deep dives in the PRE period (Figure 7). One of the whales subjected to a second exposure continued NDD during LFAS.

#### MFAS

A total of four MFAS exposures were conducted to four different whales. Three conducted NDD during MFAS exposure. The other (sw141a) was conducting relatively shallow foraging dives in the PRE period, and initiated a normal deep dive during MFAS (Figure 7).

#### Silent control

Only two silent exposures were conducted with two different sperm whales in two different sessions, both with NDD in the PRE period. One of the whales (sw141a) made an unusually-shallow deep dive during Silent Exposure, while the other continued NDD (Figure 7).

## Discussion

In this study, we investigated three species with very different natural dive patterns. These differences indicate spatial niche separation in their foraging strategies. Killer whales spend 90% of their time in the upper 20 m, and hardly ever dive deeper than 100 m (Figures 1, 2 and 5), while sperm whales spend 80% of their time diving deeper than 20 m, with foraging dives ranging from 150 to 1500 m (Figures 1, 4 and 7). Long-finned pilot whales also spend most of their time close to the surface but typically conducted bouts of foraging dives to intermediate depths of 300–600 m (Figures 1, 3 and 6).

When in DD mode at sonar onset, both killer and long-finned pilot whales apparently reacted to LFAS transmission by switching to ShD. Based on recordings of echolocation clicks and tail slap sounds on the tag record (Miller et al., 2011), such deep dives are likely to be foraging dives. Shallow dives on the other hand did not have any such vocalizations associated (Miller et al., 2011) and these dives are therefore likely not associated with feeding. A change from deep to ShD during LFAS exposure are thus likely to involve cessation of feeding. Shifts from deep to ShD for both killer- and pilot whales were associated with a general increase in maximum depth and duration of shallow dives. This indicates that shallow dives have different purposes in different behavioral modes. During periods of deep foraging dives, shallow dives may be driven by respiratory needs. When the animal is in ShD mode, as when traveling, the depth and duration of the dives are likely optimized for energy-efficient swimming and there might be an energetic (e.g., hydrodynamic) benefit of diving somewhat deeper to avoid the surface drag.

Individuals of both species that were in ShD mode at LFAS onset generally continued ShD without any change in dive behavior. Tag recordings from these shallow dives did not record echolocation or tail slaps (Miller et al., 2011), indicating shallow mode to be associated with travel or resting rather than feeding. However, if the response of the animals to sonar is to travel away from the sonar source, as described by Miller et al. (submitted) for several of these whales, continuing to travel could also be considered a response, especially if directed away from the source (Miller et al., 2011).

The reaction to MFAS was somewhat different for killer and long-finned pilot whales. Long-finned pilot whales conducted normal, deep dives during MFAS exposure (Figure 6) with recordings of foraging vocalization (Miller et al., 2011), indicating them to be foraging dives. For killer whales, one individual (327s) was in DD mode at onset of MFAS sonar, and these dives were confirmed as foraging dives by tag recording of echolocation and tail slaps in the PRE period as well as visual observations of feeding at the surface (Miller et al., 2011). The whale kept making deep dives until halfway into the exposure, before changing to ShD. The deep dives performed in the first half of the exposure lacked foraging sounds on the tag record (Miller et al., 2011), indicating cessation of feeding.

For killer and long-finned pilot whales, changes in diving behavior were most likely to occur if the animals were conducting deep foraging dives at exposure onset. Those whales that changed their dive behavior were not always those subject to the highest received sound pressure levels (Miller et al., 2011), indicating that behavioral mode may be as important as sonar exposure level in determining whether the animal will change its diving behavior or not. These findings agree with some previous studies of other cetaceans. Wartzok et al. (2004) showed that whether belugas (*Delphinapterus leucas*) responded to anthropogenic noise depended more on their activity and motivation rather than sound exposure level. Right whales (*Eubalaena glacialis)* exposed to alerting stimuli interrupted foraging dives (Nowacek et al., 2004), and killer whales changed from feeding to traveling in response to ship noise (Lusseau, 2009). Bowhead whales (*Balaena mysticetus*) differed in response to seismic shooting depending on whether the whales were feeding (Miller et al., 2005) or migrating (Richardson et al., 1999).

Sperm whales continued to dive deep during LFAS, but these dives appeared to be unusually shallow compared to dives in the PRE period (Figure 7). Deep dives during LFAS exposure were also reported to rarely contain any vocal activity (Miller et al., 2011), indicating they were not foraging dives. During MFAS exposure, sperm whales made NDD for animals in this area (Teloni et al., 2008) with vocalizations associated with feeding, indicating normal foraging activity (Miller et al., 2011). The changes in dive behavior of sperm whales during LFAS transmission agree with previous studies showing sperm whales to silence during exposure of continuous low frequency (~50 Hz) transmission (Bowles et al., 1994), as well as pingers of higher frequency (6–13 kHz) (Watkins and Schevill, 1977), while Madsen et al. (2002a) found no effect on vocal behavior of sperm whales in response to seismic surveys. Studies of sperm whale diving behavior in response to seismic air guns showed one case of abnormally long resting behavior near the sea surface during exposure at high SPL (Miller et al., 2009), and Madsen et al. (2002c) suggested variations in received levels as explanation for variations in vocal responses to anthropogenic noise.

### Biological implications of changes in dive behavior

All three species showed examples of changes in dive behavior during LFAS exposure and for killer whales also during MFAS exposure, which imply disruption of feeding activity. The change in diving was often associated with reduced echolocation vocalizations or complete silencing (Miller et al., 2011), confirming feeding cessation. Lost feeding opportunities could have significant biological effects depending on food availability and the duration of the exposure. This might be particularly severe for killer whales which feed on distinct food patches such as herring schools (Simila et al., 1996; Nøttestad et al., 2002), which might be lost if feeding is disrupted. Pilot (Weilgart and Whitehead, 1990) and sperm whales (Watwood et al., 2006) depend on sound production for prey localization and capture as they forage at depth. Both the LFAS and MFAS signals overlap in frequency with some of the foraging sounds of killer whales (Hoezel and Osborne, 1986; Ford, 1989), long-finned pilot whales (Taruski, 1979; Weilgart and Whitehead, 1990) and sperm whales (Madsen et al., 2002b,c). Therefore, sonar signals might disturb important communication between individuals during feeding, but masking of the calls will be limited because of the low duty cycle (5%) of the sonar. All three species showed stronger changes during LFAS than MFAS signals. This may be due to the difference in frequency, or it may be explained by the higher source level of the LFAS signals.

It has been proposed that a change in dive behavior in response to sonar could increase the end dive N_2_ levels and risk of tissue bubble formation in cetaceans (Jepson et al., 2003). How the observed changes in dive behavior modify this risk is not obvious. Using a previously published model (Fahlman et al., 2006) on the same dataset as described here, Kvadsheim et al. (2012) investigated how the observed changes in behavior alter the end-dive N_2_ levels, and thereby the risk of DCS. They found that the shallower DD seen in sperm whales implied an increased risk, but the change in risk was still within the normal risk range for this species. This agrees with the results in stranded animals that indicate a higher prevalence of bubbles with a gas composition associated with decompression stress in deep divers such as sperm whales and beaked whales (Bernaldo de Quiros, submitted). The changes from deep to ShD mode and the shallow dives becoming deeper seen in killer whales and long-finned pilot whales resulted in reduced risk of DCS (Kvadsheim et al., 2012). Other theoretical studies have suggested that repetitive ShD might involve an increased risk (e.g., Zimmer and Tyack, 2007). Even if the dives were deeper they were still quite shallow (>30 m) and therefore probably still within the depth zone were N_2_ is removed from the body.

Killer whales and long-finned pilot whales appear to show less changes in diving behavior during LFAS and MFAS exposure sessions during traveling mode compared to feeding mode. However, if the response of the animals to sonar is to travel away from the sonar source, as described by Miller et al. (2011), continuing to travel could also be considered a response, especially if directed away from the source (Miller et al., 2011). However, a change in travel direction during a continuous traveling state may not lead to any changes in diving behavior, the behavior examined here.

For all three species in DD mode, initial exposure to LFAS altered diving mode, resulting in likely feeding cessation or lack of conducting foraging dives. Whales subjected to a second LFAS exposure even in deep dive mode did not consistently alter their behavior, implying that there might be some habituation to the sonar in some cases.

The observed change in behavior would not result in any detrimental effects for the exposure experiments presented here, lasting only ~30 min. However, an authentic naval exercise may involve much longer periods of sonar exposure, e.g., 24 h of continuous sonar transmission (Friedman, 2006; Ainslie, 2010) or several days in the case of large international fleet exercises, and if foraging dives are not performed throughout this period, and such events are frequent, the effect will be much more severe.

### Methodological considerations

The costly and complex logistical requirements for conducting experiments like those in the current study highly limit the number of replicates. The sample size for each species, experimental condition and behavioral mode, therefore will be low. Small sample sizes are prone to risk of conducting type II statistical errors; i.e., accepting a null hypothesis when it should be rejected. Additionally, the need for testing each species, exposure type behavioral mode will need many similar tests to be conducted. Such multiple testing results in a high risk of conducting type I statistical error, i.e., obtaining a significant change when there in fact is none due to the multiple testing. Such statistic would hence not be very reliable. Therefore, a descriptive approach have been taken instead, by presenting our results more as a case-by-case interpretation for each individual exposure, by taking into account the different parameters dive mode, depth, duration, and dive rate and comparing data during each exposure session to the animal's own baseline (the PRE period).

Two of the 12 experiments with killer whales were conducted in winter [327 s (MFAS) and 317 s (LFAS)], the reminder in summer. Dive depth may vary between seasons due to variations in distribution depth of prey, and feeding dives recorded in this study were deeper in summer compared to in winter. Feeding killer whales were exposed once in winter to MFAS and once in summer to LFAS, with the LFAS summer experiment resulting in an abrupt cessation of feeding and change to ShD early in the sonar exposure session, while in the winter MFAS experiment the whales continued DD and feeding at least halfway into the exposure. However, it cannot be determined whether it was the difference in transmission types, seasonal differences, or other sources of variability of responsiveness that cause the difference in response onset.

Some animals were subject to two exposures of the same type (Figures 5–7). The response to the second exposure may thus be influenced by the animals prior experience with this signal, causing either a sensitization or habituation, depending on whether the signal was perceived as a real threat or not. Killer whales 144a and 144b did not resume deep foraging dives after the first LFAS exposure, and the observed lack of response to the second exposure may therefore be influenced by the animals already being in travel mode. In contrast, both pilot and sperm whales tended to resume deep foraging dives between exposure sessions. Long-finned pilot whales 138a and 138b were conducting shallow travel dives at onset of the first LFAS exposure, continuing this behavior, while in the second exposure they were conducting deep foraging dives at LFAS onset, but then switched to shallow travel dives. Pilot whale 157d was conducting deep foraging dives at onset of the first LFAS exposure, switching to shallow travel dives, while during the second LFAS exposure the whale was conducting ShD, and continued this. However, pilot whale 156b was conducting deep foraging dives prior to both LFAS exposures, but switched to shallow travel dives only for the first exposure. Both of the sperm whales subject to multiple LFAS exposures were conducting normal deep foraging dives prior to all LFAS exposures. They both responded by switching to unusual shallow deep dives without foraging during the first LFAS, and this was also the case for the second exposure for sperm whale 142a for the second exposure. Sperm whale 160a did however continue NDD in the second exposure. The present result does not show any clear signs of habituation or sensation, but one should however always treat the second exposure with care.

## Conclusions

The present study have shown that killer whales that are feeding at onset of exposure may change their diving behavior by switching from deep feeding dives to shallow travel dives when exposed to LFAS (1–2 kHz) and MFAS (6–7 kHz) naval sonar signals. When traveling at sonar onset however, no changes in dive behavior were found. Long-finned pilot whales and sperm whales performed normal deep foraging dives during MFAS exposure, while during LFAS exposures, long-finned pilot whales performed fewer deep foraging dives and some sperm whales performed shallower and shorter dives without foraging.

### Conflict of interest statement

This study is mainly funded by three naval organizations; The US Office of Naval Research, The Norwegian Ministry of Defense, and the Dutch Ministry of Defense. In addition the Norwegian Research Council and WWF-Norway have also contributed financially. Funders had no role in study design, data analysis or preparation of the manuscript. Authors are employed by government (L. D. Sivle, P. H. Kvadsheim)-, independent non-profit (A. Fahlman, P. L. Tyack, and F. P. A. Lam)- or academic (A. Fahlman, P. L. Tyack, and P. J. O. Miller) research organizations. No authors are employed by naval organizations.

## References

[B2] Ainslie M. A. (2010). Principles of Sonar Performance Modeling. Chichester, UK: Springer-Praxis

[B3] Bernaldo de Quirós Y. Gonzales-Diaz O. Arbelo M. Sierra E. Sacchini S. Fernández A. (2012). Decompression versus decomposition: distribution, quantity and gas composition of bubbles in stranded marine mammals. Front. Physiol. 3:177 10.3389/fphys.2012.00177 22675306 PMC3366475

[B4] Bowles A. E. Smultea M. Wursig B. Demaster D. P. Palka D. (1994). Relative abundance and behavior of marine mammals exposed to transmissions from the Heard Island feasibility test. J. Acoust. Soc. Am. 96, 2469–2484 10.1121/1.410120 7963037

[B5] Cox T. M. Ragen A. J. Vos E. Baird R. W. Balcomb K. Barlow J. (2006). Understanding the impact of anthropogenic sound on beaked whales. J. Cetacean Res. Manage. 7, 177–187

[B6] D'Amico A. D. Gisiner R. Ketten D. R. Hammock J. A. Johnson C. Tyack P. L. (2009). Beaked whale strandings and naval exercises. Aquat. Mamm. 35, 452–472

[B7] Fahlman A. Olszowka A. Bostrom B. Jones D. R. (2006). Deep diving mammals: dive behavior and circulatory adjustments contribute to bends avoidance. Respir. Physiol. Neurobiol. 153, 66–77 10.1016/j.resp.2005.09.014 16413835

[B8] Ford J. K. B. (1989). Acoustic behavior of resident killer whales (*Orcinus orca*) off Vancouver Island, British Columbia. Can. J. Zool. 67, 727–745 10.1121/1.1349537 11303937

[B9] Frantzis A. (1998). Does acoustic testing strand whales? Nature 392, 29 10.1038/32068 9510243

[B10] Friedman N. (2006). The Naval Institute Guide to World Naval Weapon Systems (Naval Institute Press, Annapolis). Annapolis, MD: Naval Institute Press

[B12] Hoezel A. R. Osborne R. W. (1986). Killer whale call characteristics: implications for cooperative foraging strategies, in Behavioral Biology of Killer Whales, eds Kirkevold B. C. Lockard J. C. (New York, NY: Alan, R. Liss Inc.), 373–403

[B14] Jepson P. D. Arbelo M. Deaville R. Patterson I. A. P. Castro P. Baker J. R. (2003). Gas-bubble lesions in stranded cetaceans-Was sonar responsible for a spate of whale deaths after an Atlantic military exercise? Nature 425, 575–576 10.1038/425575a 14534575

[B15] Johnson M. Tyack P. L. (2003). A digital acoustic recording tag for measuring the response of wild marine mammals to sound. IEEE J. Ocean Eng. 28, 3–12

[B16] Kastelein R. A. Verboom W. C. Jennings N. De Haan D. (2008a). Behavioral avoidance threshold level of a harbor porpoise (*Phocoena phocoena*) for a continuous 50 kHz pure tone (L). J. Acoust. Soc. Am. 123, 1858–1861 10.1121/1.2874557 18396994

[B17] Kastelein R. A. Verboom W. C. Jennings N. De Haan D. Van Der Heul S. (2008b). The influence of 70 and 120 kHz tonal signals on the behavior of harbor porpoises (*Phocoena phocoena*) in a floating pen. Mar. Environ. Res. 66, 319–326 10.1016/j.marenvres.2008.05.005 18599117

[B19] Kramer D. L. (1988). The behavioural ecology of air breathing by aquatic animals *Can*. J. Zool. 66, 89–94

[B20] Kvadsheim P. H. Lam F. P. A. Miller P. J. O. Alves A. C. Antunes R. Bocconcelli A. (2009). Cetaceans and naval sonar - the 3S-2009 cruise report, in FFI-Rapport 2009/01140. Forsvarets Forskningsintitutt.

[B21] Kvadsheim P. H. Miller P. J. O. Tyack P. Sivle L. D. Lam F. P. A. Fahlman A. (2012). Estimated tissue and blood N2 levels and risk of decompression sickness in deep-, intermediate and shallow diving toothed whales during exposure to naval sonar. Front. Aquat. Physiol. 3:125 10.3389/fphys.2012.00125 22590458 PMC3349243

[B22] Lusseau D. (2009). Vessel traffic disrupts the foraging behavior of southern resident killer. whales. Endang. Species Res. 6, 211–221

[B23] Madsen P. T. Møhl B. Nielsen B. K. Wahlberg M. (2002a). Male sperm whale behavior during exposure to distant seismic survey pulses. Aquat. Mamm. 28, 231–240

[B24] Madsen P. T. Payne R. Kristiansen N. U. Wahlberg M. Kerr I. Mohl B. (2002b). Sperm whale sound production studied with ultrasound time/depth-recording tags. J. Exp. Biol. 205, 1899–1906 12077166 10.1242/jeb.205.13.1899

[B25] Madsen P. T. Wahlberg M. Mohl B. (2002c). Male sperm whale (*Physeter macrocephalus*) acoustics in a high-latitude habitat: implications for echolocation and communication. Behav. Ecol. Sociobiol. 53, 31–41

[B28] Miller G. W. Moulton V. D. Davis R. A. Holst M. Millman P. MacGillivray A. O. (2005). Monitoring seismic effects on marine mammals- southeastern Beaufort Sea, 2001–2002, in Offshore oil and Gas Environmental Effects Monitoring: Approaches and Technologies, eds Armsworthy S. L. Cranford P. J. Lee K. (Columbus, OH: Battelle Press), 511–542

[B31] Miller P. J. O. Antunes R. Alves A. C. Wensveen P. Kvadsheim P. H. Kleivane L. (2011). The 3S experiments: studying the behavioral effects of sonar on killer whales (*Orcinus orca*), sperm whales (*Physeter macrocephalus*), and long-finned pilot whales (*Globicephala melas*) in Norwegian waters. Scottich Ocean Inst. Tech. Rep. 2011-001, 289

[B30] Miller P. J. O. Johnson M. P. Madsen P. T. Biassoni N. Quero M. Tyack P. L. (2009). Using at-sea experiments to study the effects of airguns on the foraging behavior of sperm whales in the Gulf of Mexico. Deep Sea Res. Part I Oceanogr. Res. Pap. 56, 1168–1181

[B32] Miller P. J. O. Shapiro A. D. Deecke V. B. (2010). The diving behavior of mammal-eating killer whales (*Orcinus orca*): variations with ecological not physiological factors. Can. J. Zool. 88, 1103–1112

[B35] Morton A. B. Symonds H. K. (2002). Displacement of *Orcinus orca* (L.) by high amplitude sound in British Columbia, Canada. ICES J. Mar. Sci. 59, 71–80

[B36] Nøttestad L. Ferno A. Axelsen B. E. (2002). Digging in the deep: killer whales' advanced hunting tactic. Polar Biol. 25, 939–941

[B37] Nowacek D. P. Johnson M. P. Tyack P. L. (2004). North Atlantic right whales (*Eubalaena glacialis*) ignore ships but respond to alerting stimuli. Proc. R. Soc. Lond. B Biol. Sci. 271, 227–231 10.1098/rspb.2003.2570 15058431 PMC1691586

[B38] Nowacek D. P. Thorne L. H. Johnston D. W. Tyack P. L. (2007). Responses of cetaceans to anthropogenic noise. Mamm. Rev. 37, 81–115

[B39] Olesiuk P. F. Nichol L. M. Sowden M. J. Ford J. K. B. (2002). Effect of the sound generated by an acoustic harassment device on the relative abundance and distribution of harbor porpoises (*Phocoena phocoena*) in retreat passage, British Columbia. Mar. Mammal Sci. 18, 843–862

[B42] Richardson W. J. Greene C. R. Malme C. I. Thomson D. H. (1995). Marine Mammals and Noise. San Diego, CA: Academic press

[B43] Richardson W. J. Miller G. V. Greene C. R. (1999). Displacement of migrating bowhead whales by sounds from seismic surveys in shallow waters of the Beaufort Sea. J. Acous. Soc. Am. 106, 2281

[B45] Sibley R. M. Nott H. M. R. Fletcher D. J. (1990). Splitting behavior into bouts. Anim. Behav. 39, 63–69

[B46] Simila T. Holst J. C. Christensen I. (1996). Occurrence and diet of killer whales in northern Norway: seasonal patterns relative to the distribution and abundance of Norwegian spring-spawning herring. Can. J. Fish. Aquat. Sci. 53, 769–779

[B47] Simmonds M. P. Lopez-jurado L. F. (1991). Whales and the military. Nature 351, 448

[B50] Slater P. J. B. Lester N. P. (1982). Minimizing errors in splitting behavior into bouts. Behaviour 79, 153–161

[B52] Taruski A. G. (1979). The whistle repertoire of the North Atlantic pilot whales (*Globicephala melas*) and its relationship to behavior and environment, in Behaviour of Marine Animals, eds Winn H. E. Olla B. L. (New York, NY: Plenum Press), 345–368

[B53] Teloni V. Johnson M. P. Miller P. J. O. Madsen P. T. (2008). Shallow food for deep-divers: dynamic foraging behavior of male sperm whales in a high latitude habitat. J. Exp. Mar. Biol. Ecol. 354, 119–131

[B54] Tyack P. L. (2008). Implications for marine mammals of large-scale changes in the marine acoustic environment. J. Mammal. 89, 549–558

[B56] Watkins W. A. Schevill W. E. (1977). Spatial distribution of *Physeter catodon* (Sperm Whales) underwater. Deep Sea Res. Part I-Oceanogr. Res. Pap. 27, 693–699

[B57] Wartzok D. Popper A. Gordon J. Merrill J. (2004). Factors affecting the responses of marine mammals to acoustic disturbance. Mar. Technol. Soc. J. 37, 6–15

[B58] Watwood S. L. Miller P. J. O. Johnson M. Madsen P. T. Tyack P. L. (2006). Deep diving foraging behavior of sperm whales (*Physeter macrocephalus*). J. Anim. Ecol. 75, 814–825 10.1111/j.1365-2656.2006.01101.x 16689963

[B59] Weilgart L. S. Whitehead H. (1990). Vocalizations of the north-Atlantic pilot whale (*Globicephala melas*) as related to behavioural contexts. Behav. Ecol. Sociobiol. 26, 399–402

[B60a] Zimmer W. M. X. Tyack P. L. (2007). Repetitive shallow dives pose decompression risk in deep diving beaked whales. Mar. Mamm. Sci. 23, 888–925

